# Exploring the psychological landscape of thyroid nodules: resilience, anxiety, and ultrasound correlations

**DOI:** 10.3389/fpsyg.2025.1567391

**Published:** 2025-06-25

**Authors:** Jing Wang, Lixia Wang, Daidi Zhang, Guoqing Chen, Qinfang Zhang, Haiyan Chen, Jiamao Cheng

**Affiliations:** ^1^Department of Ultrasound Medicine, First Affiliated Hospital of Dali University, Dali, Yunnan, China; ^2^School of Basic Medicine, Dali University, Dali, Yunnan, China; ^3^School of Clinical Medicine, Dali University, Dali, Yunnan, China; ^4^Department of Neurology, First Affiliated Hospital of Dali University, Dali, Yunnan, China

**Keywords:** thyroid nodules, ultrasonic characteristics of C-TIRADS, resilience, anxiety, correlations

## Abstract

**Background:**

While thyroid nodules (TN) represent a prevalent clinical entity with ultrasound-guided management paradigms, the psychological sequelae of diagnostic imaging remain underexplored. This study investigates the complex interplay between ultrasound characteristics (C-TIRADS), psychological resilience, and anxiety manifestations in TN patients, addressing a critical gap in psychosomatic thyroidology.

**Methods:**

In this prospective cross-sectional study, 303 consecutive TN patients (mean age 47.3 ± 12.1 years, 72.6% female) underwent standardized ultrasound evaluation using Aplio500 systems (Toshiba) at the First Affiliated Hospital of Dali University (October 2022–October 2024). C-TIRADS classifications were independently adjudicated by two radiologists. Psychological assessments employed the Hamilton Anxiety Rating Scale (HAMA) and Connor-Davidson Resilience Scale (CD-RISC) within 48 h post-examination. Advanced statistical analyses included: (1) Multivariate regression modeling accounting for demographic confounders. (2) Spearman/Kendall correlation matrices. (3) Ordinal logistic regression for malignancy risk stratification.

**Results:**

Psychological assessments revealed that TN^+^ patients exhibited elevated resilience (CD-RISC total: 64.04 ± 14.166 vs. 60.61 ± 15.074; *p* = 0.025) but paradoxically higher anxiety levels (HAMA total: 32.51 ± 8.516 vs. 30.67 ± 8.667; *p* = 0.005), demonstrating a negative correlation between resilience and anxiety severity (*r* = −0.259, *p* < 0.001). Ultrasonographic analysis demonstrated significant psychosomatic associations: Higher C-TIRADS classifications predicted reduced psychological resilience (OR = 0.327, 95%CI = 0.114–0.943, *p* = 0.044) and increased nodule multiplicity (OR = 0.135, 95%CI = 0.034–0.537, *p* = 0.005). Specific ultrasound features showed differential anxiety impacts – irregular margins increased anxiety risk (OR = 362.080, *p* = 0.037) while capsular protrusion showed protective effects (OR = 0.003, *p* = 0.028). Symptom correlation analyses revealed: (1) Somatic anxiety showed stronger cardiovascular/respiratory system associations (*r* = 0.703–0.704). (2) Psychic anxiety correlated with cognitive-emotional domains (tension: *r* = 0.795; insomnia: *r* = 0.740). (3) Anxiety dimensions demonstrated differential resilience impacts – somatic anxiety primarily affected optimism (*r* = −0.146, *p* = 0.011), while psychic anxiety impaired overall resilience (*r* = −0.248, *p* < 0.001).

**Conclusion:**

Higher C-TIRADS malignancy risk classifications were associated with reduced resilience and increased anxiety, particularly in patients with irregular nodule edges. Clinical approaches should focus on psychological support to boost resilience, treatment outcomes, and quality of life.

## Introduction

Thyroid nodules (TN) are frequently encountered in clinical practice, with the majority being benign. However, accurately distinguishing between benign and malignant nodules is crucial for determining appropriate treatment strategies (Richter et al., 2024). Currently, the primary clinical diagnostic methods include ultrasound imaging, fine needle aspiration biopsy, and serological testing. Ultrasound serves as the primary modality for the initial evaluation of thyroid nodules, aiding in risk stratification by assessing the nodules’ size, shape, and echogenic characteristics (Giovanella et al., 2024). The Chinese Thyroid Imaging Reporting and Data System (C-TIRADS) is a tool designed to evaluate the malignancy risk of thyroid nodules, with classification criteria based on imaging features such as size, shape, margins, and echogenicity (Wu et al., 2024). Research indicates that C-TIRADS exhibits varying diagnostic efficacy depending on the size of the TN, with optimal performance observed in nodules measuring 1–4 cm, effectively differentiating between benign and malignant cases (Yang et al., 2024). Furthermore, the C-TIRADS system integrates various factors, including growth patterns, microcalcifications, blood flow characteristics, and other multimodal ultrasound features, to construct a predictive model that enhances its clinical applicability (Wu et al., 2024). When combined with contrast-enhanced ultrasound and other emerging technologies, this approach is intended to improve diagnostic accuracy and reduce the incidence of unnecessary biopsies (Cao et al., 2022). However, the diagnosis of thyroid nodules can provoke a spectrum of emotional responses in patients, and effectively addressing and managing these emotional reactions remains a significant challenge in current research.

The diagnosis of thyroid nodules frequently elicits emotional responses in patients, including fear, anxiety, and shock, which are significantly influenced by the individual’s psychological resilience and rational risk assessment (Naunheim et al., 2023). Patients exhibiting high levels of resilience typically demonstrate a greater capacity to manage the uncertainty and stress associated with the diagnostic process, thereby mitigating symptoms of anxiety and depression (Kurtses Gürsoy and Köseoğlu Toksoy, 2023). Research indicates a correlation between the psychological state of patients with thyroid nodules and the ultrasound characteristics of these nodules. During the COVID-19 pandemic, there was a notable increase in negative emotions, with anxiety showing a positive correlation with specific ultrasound features of thyroid nodules, such as microcalcifications, hypoechoic nodules, and irregular nodule margins (Lei et al., 2023). Additionally, larger thyroid nodules may be associated with heightened anxiety levels (Kornelius et al., 2020). Risk factors for thyroid cancer, including microcalcifications and irregular nodule shapes, are closely linked to the anxiety levels experienced by patients (Huang et al., 2021). This study seeks to examine the relationship between C-TIRADS ultrasound characteristics and patients’ psychological resilience and anxiety. It also aims to explore the correlation between psychological resilience and the characteristics of thyroid nodules, as well as the anxiety experienced by patients upon learning about the ultrasonic features of their thyroid nodules.

## Materials and methods

### Study design and sample

This cross-sectional study recruited 303 thyroid nodule (TN^+^) patients and 126 controls (TN^−^) from October 2022 to October 2024, who had been diagnosed with thyroid nodules at the First Affiliated Hospital of Dali University. After conducting the initial ultrasonic examination of the thyroid nodules, the assessment was completed within 30 min using the evaluation system developed by Beta Psychology (China, First Affiliated Hospital of Chongqing Medical University). A total of 305 evaluation plans were distributed, all of which were returned, resulting in 303 valid responses. Two responses were excluded due to incomplete inspection results, yielding a recovery rate of 100% and an effective rate of 99.3%. The sample comprised 38 males and 265 females. Among the participants, 201 exhibited abnormal resilience, representing 66.3% of the sample, while 177 individuals were identified as experiencing anxiety, accounting for 58.4%. The inclusion criteria for the study were as follows: (1) participants must be 18 years of age or older; (2) a Color Doppler ultrasound must have been conducted at our hospital, with complete and comprehensive medical history, ultrasound images, and evaluation forms; (3) participants must have no history of mental illness, possess independent behavioral capabilities, and provide voluntary consent to participate in the study. (4) Resilience and anxiety levels were categorized using validated cutoffs for the CD-RISC (abnormal resilience: score < 60) and HAMA (anxiety: score ≥ 8). Additionally, a control group consisting of 126 patients with thyroid nodules classified as TN (−) was also established. This research met the ethical requirements and was approved by the ethics committee of the First Affiliated Hospital of Dali University in Yunnan, China (ethics number: DFY20250126001).

### Study instruments and methods

The Aplio500 intelligent Color Doppler Flow Imaging (CDFI) diagnostic instrument was utilized, with a probe frequency range of 7–12 MHz. Patients were positioned supine with their necks fully exposed for optimal examination. Thyroid nodules were assessed using multiple sectional views, and their ultrasonic characteristics were documented in accordance with the C-TIRADS classification system. Ultrasound images were independently interpreted by an attending physician and a deputy chief physician, possessing 5 and 10 years of experience in the ultrasound department, respectively. In instances of disagreement between the two physicians, a chief physician with over 15 years of experience in ultrasound diagnostics provided the final evaluation. In the initial section of the study, we examined the differences in the characteristics of the study cohort, as well as the Hamilton Anxiety Rating Scale (HAMA) and Connor-Davidson Resilience Scale (CD-RISC) factors, between the TN^−^ and TN^+^ groups, identifying statistically significant indicators. In the second section, we employed ordinal logistic regression to analyze the interaction between C-TIRADS ultrasound characteristics and HAMA and CD-RISC factors within the TN^+^ group.

### Statistical analysis

The obtained data were analyzed using SPSS software version 29.0 (IBM, New York, United States). The continuous variables of normal distribution such as age, body mass index, cd-risc total score and resilience were described by means of mean and standard deviation (SD), and the continuous variables were compared by student’s *t*-test. The non-normal distribution variables are expressed as the quartile range of the median and the 25th-75th percentile. The Mann–Whitney *U* test is used for the non-normal distribution data. Gender, education level, nationality, marital status, smoking history, drinking history, hypertension, diabetes, family history of thyroid cancer, history of polycystic ovary, HAMA total score, physical anxiety, mental anxiety, results and results rating, CD-RISC strength, and other classification data were described by frequency and percentage. The classification variables were tested using the chi-square test. Ordinal logistic regression was used to analyze the interaction between c-tirads ultrasound characteristics and HAMA, cd-risc factors. Binary logistic regression model was used to screen significant anxiety risk factors. Spearman correlation analysis was performed on the factors with statistical significance between HAMA and cd-risc, and Spearman and Kendall correlation analysis was performed on the factors with statistical significance between HAMA and 14 specific topics: As the absolute value of the correlation coefficient (|*r*|) approaches 1, the correlation between the two variables becomes stronger, signifying a more robust relationship. This relationship can be categorized as follows: very low correlation (0.00–0.19), low correlation (0.20–0.39), moderate correlation (0.40–0.69), high correlation (0.70–0.89), and very high correlation (0.90–1.00). This study statistically analyzed the differences in TN ultrasound characteristics, resilience, and anxiety. It further examined the correlation between resilience and anxiety in TN patients and revealed the distinct characteristics of anxiety in TN patients. Ordinal logistic regression was used to explore the interaction between C-TIRADS ultrasound characteristics and the HAMA and CD-RISC factors. A binary logistic regression model was employed to identify significant risk factors for anxiety. Spearman correlation analysis was conducted on factors showing statistical significance between HAMA and CD-RISC. Both Spearman and Kendall correlation analyses were applied to factors demonstrating statistical significance between HAMA and 14 specific topics. A two-tailed *p*-value of less than 0.05 was considered indicative of statistical significance. This study conducted a statistical analysis of the differences in TN ultrasound characteristics, resilience, and anxiety, further explored the correlation between resilience and anxiety in TN patients, and elucidated the distinct characteristics of anxiety in this patient population.

## Results

### Study cohort characteristics

Tables 1–3 provides a summary of the characteristics of the study population. A total of 429 participants were included in the study, comprising 303 individuals in the TN^+^ group and 126 individuals in the TN^−^ control group. There were no statistically significant differences between the two groups concerning unhealthy habits (such as smoking history and alcohol consumption history), family disease history (including hypertension, diabetes, thyroid cancer, and polycystic ovary syndrome), or demographic characteristics (age, BMI, gender, education level, ethnicity, and marital status) (*p* > 0.05). However, a significant difference was noted in the frequency of weekly consumption of pickled foods, with the TN^+^ group exhibiting a higher frequency compared to the TN^−^ group (*p* = 0.006). Specifically, the distribution of consumption frequencies for the TN^+^ and TN^−^ groups were as follows: 0 times (85.9% vs. 14.1%), 1–2 times (65.5% vs. 34.5%), and 3 times or more (69.5% vs. 30.5%).

**Table 1:** Outlines the demographic characteristics of the TNs^+^ and TNs^−^ groups.

Variables	Category	Thyroid nodules		*t/Z/χ* ^2^	*p*
		Absent (*n* = 126)	Present (*n* = 303)		
Age (years)		40.26 ± 11.4	41.83 ± 11.5	−1.291	0.198
Body mass index (kg/m^2^)		22.3(20.3, 24.5)	22.9(20.5, 25)	0.555	0.579
Gender (%)	Male	20(34.5)	38(65.5)	0.845	0.358
	Female	106(28.6)	265(71.4)		
Education (%)	Primary school and below	28(29.5)	67(70.5)	3.016	0.698
	Junior high school	22(28.2)	56(71.8)		
	Senior high school	25(30.5)	57(69.5)		
	Associate’s degree	14(22.2)	49(77.8)		
	Bachelor’s degree	36(34)	70(66)		
	Postgraduate	1(20)	4(80)		
Nationality (%)	Han	52(31.3)	114(68.7)	7.638	0.359
	Bai	48(28.2)	122(71.8)		
	Yi	9(31)	20(69)		
	Lisu	5(27.8)	13(72.2)		
	Hui	1(8.3)	11(91.7)		
	Pumi	5(60)	4(40)		
	Naxi	1(9.1)	11(90.9)		
	Other	5(38.5)	8(61.5)		
Marital status (%)	Unmarried	36(29)	88(71)	1.462	0.753
	Married	89(29.9)	209(70.1)		
	Divorced	0(0)	4(100)		
	Widowed	1(33.3)	2(66.7)		

**Table 2:** Detrimental Behaviors of TNs^+^ and TNs^−^ Groups.

Variables	Category	Thyroid nodules (%)		*χ^2^*	*p*
		Absent (*n* = 126)	Present (*n* = 303)		
Smoking history	Never	120(29.6)	286(70.4)	0.126	0.722
	Ever	6(26.1)	17(73.9)		
Alcohol consumption history	Never	120(29.1)	292(70.9)	0.289	0.592
	Ever	6(35.3)	11(64.7)		
Consumption of preserved foods	0 times/week	10(14.1)	61(85.9)	10.27	**
	1–2 times/week	58(34.5)	110(65.5)		
	≥3 times/week	58(30.5)	132(69.5)		

**p < 0.01.

**Table 3:** Details the family history of the TNs^+^ and TNs^−^ groups.

Variables	Category	Thyroid nodules (%)		*χ^2^*	*p*
		Absent (*n* = 126)	Present (*n* = 303)		
Hypertension	No	122(29.5)	291(70.5)	0.158	0.788
	Yes	4(25)	12(75)		
Diabetes mellitus	No	123(29.3)	297(70.7)	0.068	0.726
	Yes	3(33.3)	6(66.7)		
Family history of thyroid cancer	Never	123(30)	284(70)	1.681	0.195
	Ever	4(17.4)	19(82.6)		
History of polycystic ovarian syndrome	Never	124(29.6)	295(70.4)	0.466	0.730
	Ever	2(20)	8(80)		

### The total difference in the CD-RISC and HAMA scores

The TN ^−^ cohort exhibited mean ± standard deviation scores of 60.61 ± 15.074 for the total CD-RISC and 30.67 ± 8.667 for resilience. In contrast, the TN^+^ cohort reported mean ± standard deviation scores of 64.04 ± 14.166 for the total CD-RISC and 32.51 ± 8.516 for resilience. Statistically significant differences were identified between the two groups in terms of total CD-RISC scores, resilience, and optimism (*p* = 0.025, 0.044, 0.036, respectively, as shown in Table 4). Furthermore, significant differences were observed in HAMA total scores, somatic anxiety, psychic anxiety, and outcome ratings between the groups (*p* = 0.005, 0.006, 0.019, and <0.001, respectively, as detailed in Table 4).

**Table 4:** Differential analysis of CD-RISC factor and HAMA factor between two groups.

Scale	Scale factor	Thyroid nodules		*t*/*Z*/*χ*^2^	*p*
		Absent (*n* = 126)	Present (*n* = 303)		
CD-RISC	Total score	60.61 ± 15.074	64.04 ± 14.166	−2.242	0.025
	Resilience	30.67 ± 8.667	32.51 ± 8.516	4.098	0.044
	Strength	21(17.75, 24)	22(19, 26)	−1.934	0.053
	Optimism	9(7, 11)	9(8, 11)	−2.097	0.036
	Result rating (%)	126(29.4)	303(70.6)	2.876	0.411
HAMA	Total score	14(6, 21.25)	9(5, 15)	−2.799	0.005
	Somatic anxiety	4(1, 10)	3(1, 6)	−2.771	0.006
	Psychic anxiety	8(4, 12.25)	6(4, 10)	−2.347	0.019
	Result rating (%)	126(29.4)	303(70.6)	19.719	<0.001

### The correlation between C-TIRADS ultrasound characteristics and psychological resilience needs to be further investigated

**Figure 1:**
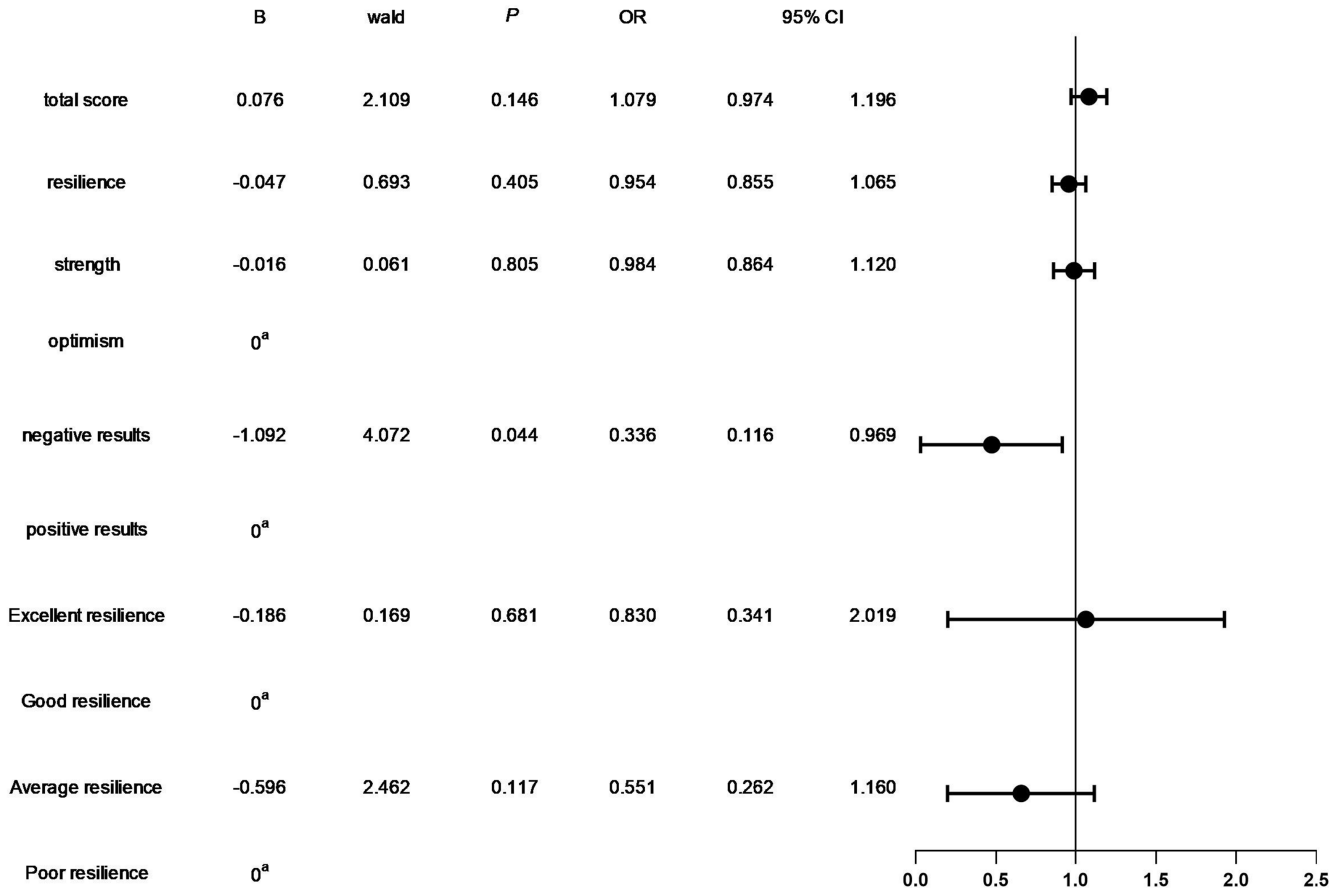
Ordered logistic regression analysis of the impact of C-TIRADS grading on psychological resilience factors.

**Figure 2:**
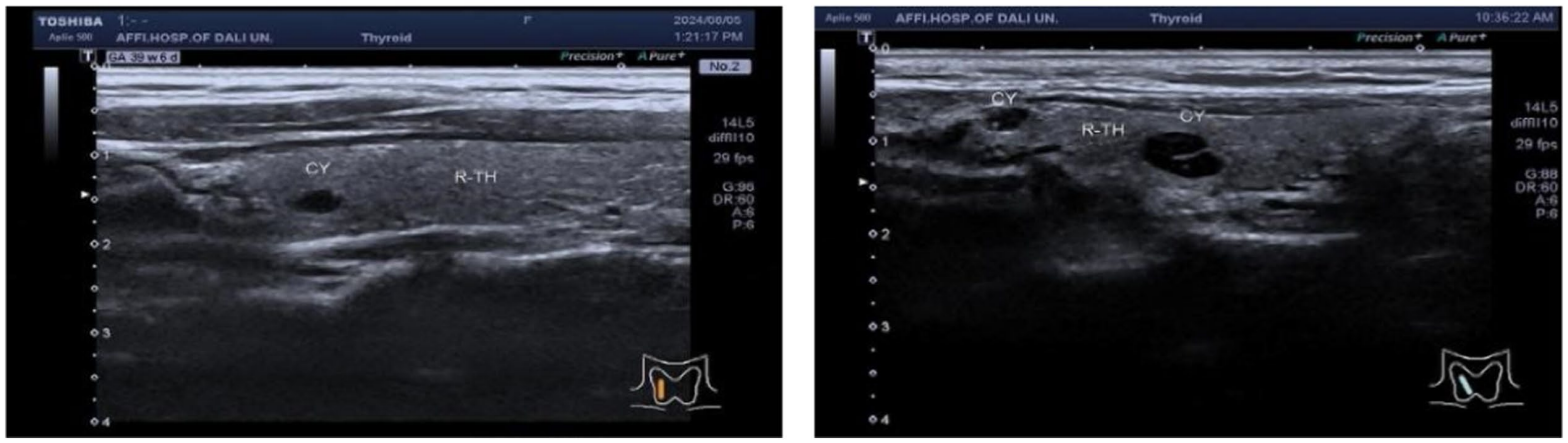
Ultrasound image of cystic nodular lesion in the right lobe of the thyroid gland, C-TIRADS classification: Class 2, multiple cystic nodular lesions in the right lobe of the thyroid gland, C-TIRADS classification: Class 2.

**Figure 3:**
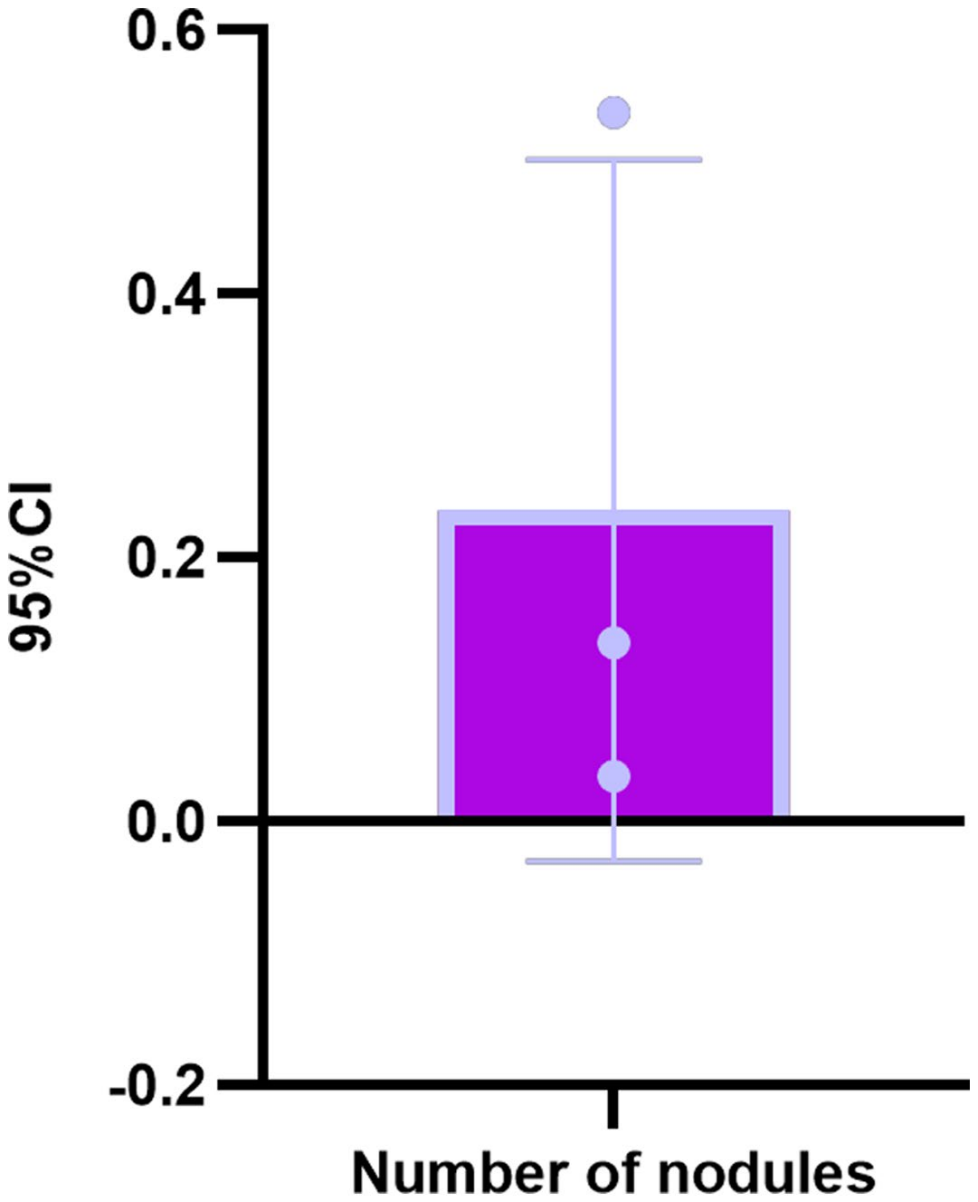
Binary logistic regression analysis of C-TIRADS classification of ultrasound characteristic nodules.

In the TN^+^ group, comprising 303 patients, the distribution of C-TIRADS classifications among the patients was as follows: 51 cases in class 2, 114 cases in class 3, 59 cases in class 4A, 33 cases in class 4B, 40 cases in class 4C, and 6 cases in class 5.Regarding resilience outcomes, 102 cases were normal, while 201 cases were abnormal, with resilience levels categorized as excellent in 42 cases, good in 60 cases, fair in 77 cases, and poor in 124 cases. The ordered logistic regression analysis revealed that the differences in resilience outcomes were statistically significant [*p* = 0.044, *B* = −1.092, OR, 95% CI = 0.327 (0.114–0.943)], indicating that higher c-TIRADS classifications were associated with lower psychological resilience, as depicted in Figure 1. Furthermore, binary logistic regression analysis demonstrated a significant difference in the number of nodules across C-TIRADS classifications [*p* = 0.005, *B* = −2.004, OR, 95% CI = 0.135 (0.034–0.537)], as illustrated in Figures 2, 3.

### The correlation between ultrasonic characteristics of C-TIRADS and anxiety states needs to be further investigated

**Figure 4:**
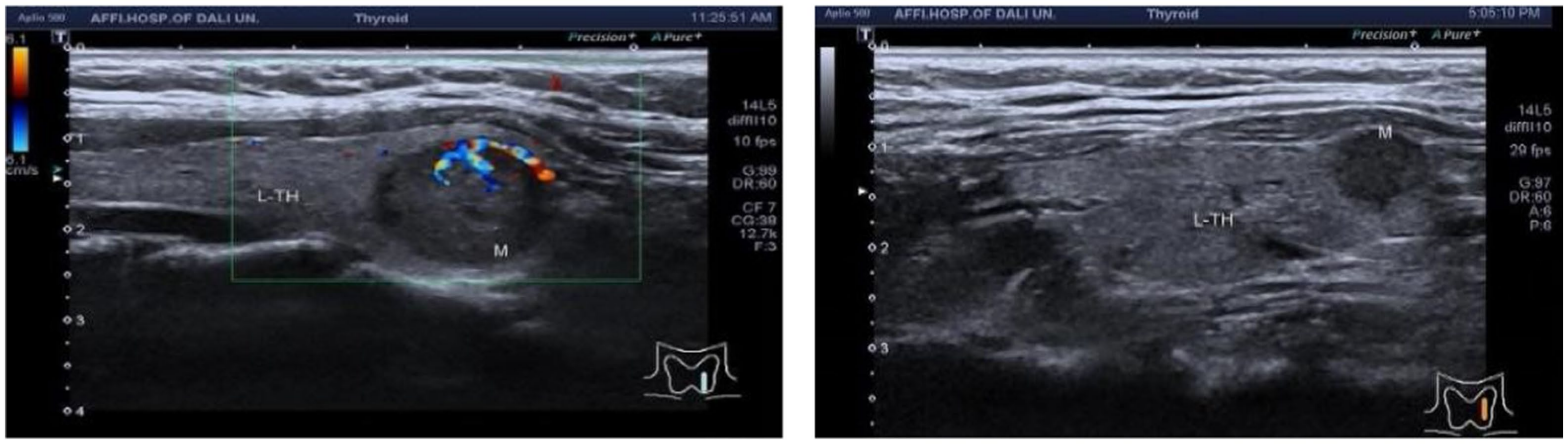
Ultrasound image of nodular lesion in the left lobe of the thyroid gland, C-TIRADS classification: 4A.

**Figure 5:**
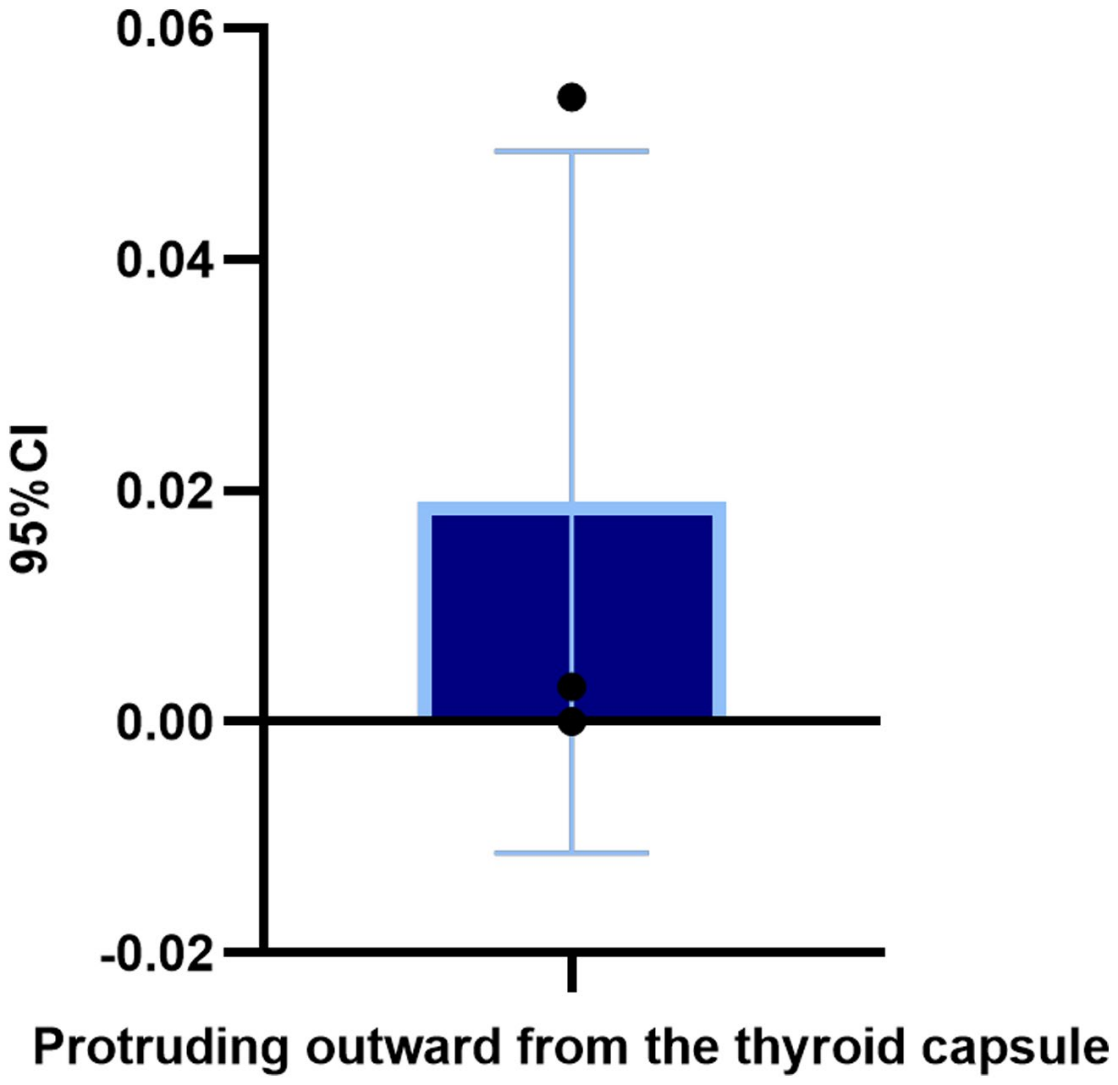
Binary logistic regression analysis of C-TIRADS classification ultrasound features to determine whether TN protrudes beyond the thyroid capsule.

**Figure 6:**
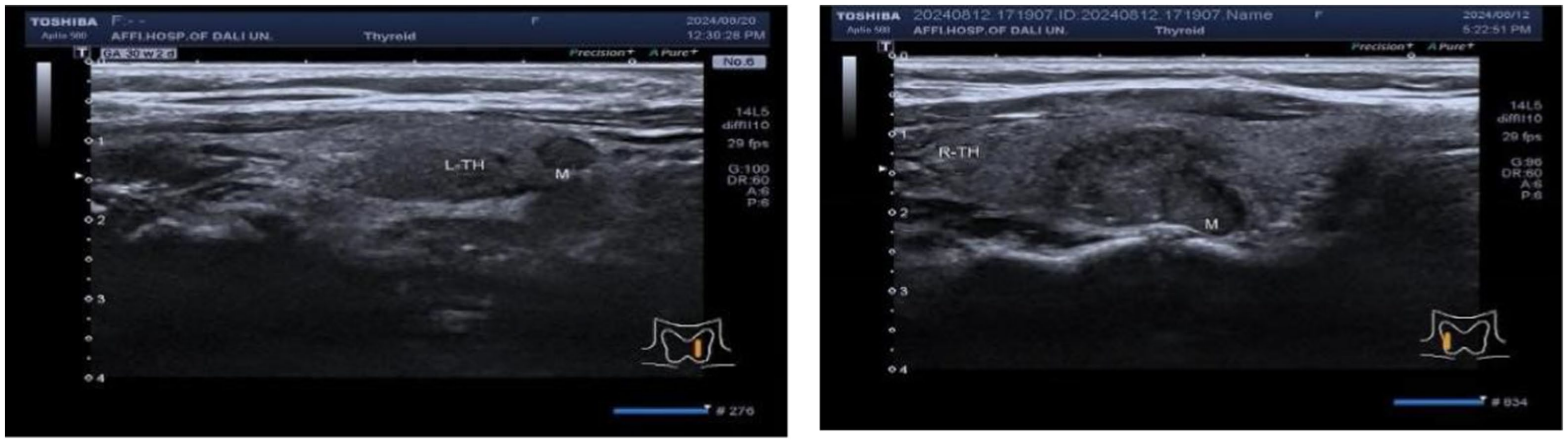
Ultrasound image of nodular lesion in the left lobe of the thyroid gland, C-TIRADS classification: 4B class, TN edge smooth, ultrasound image of nodular lesion in the right lobe of the thyroid gland, C-TIRADS classification: 4C class, TN edge not smooth.

**Figure 7:**
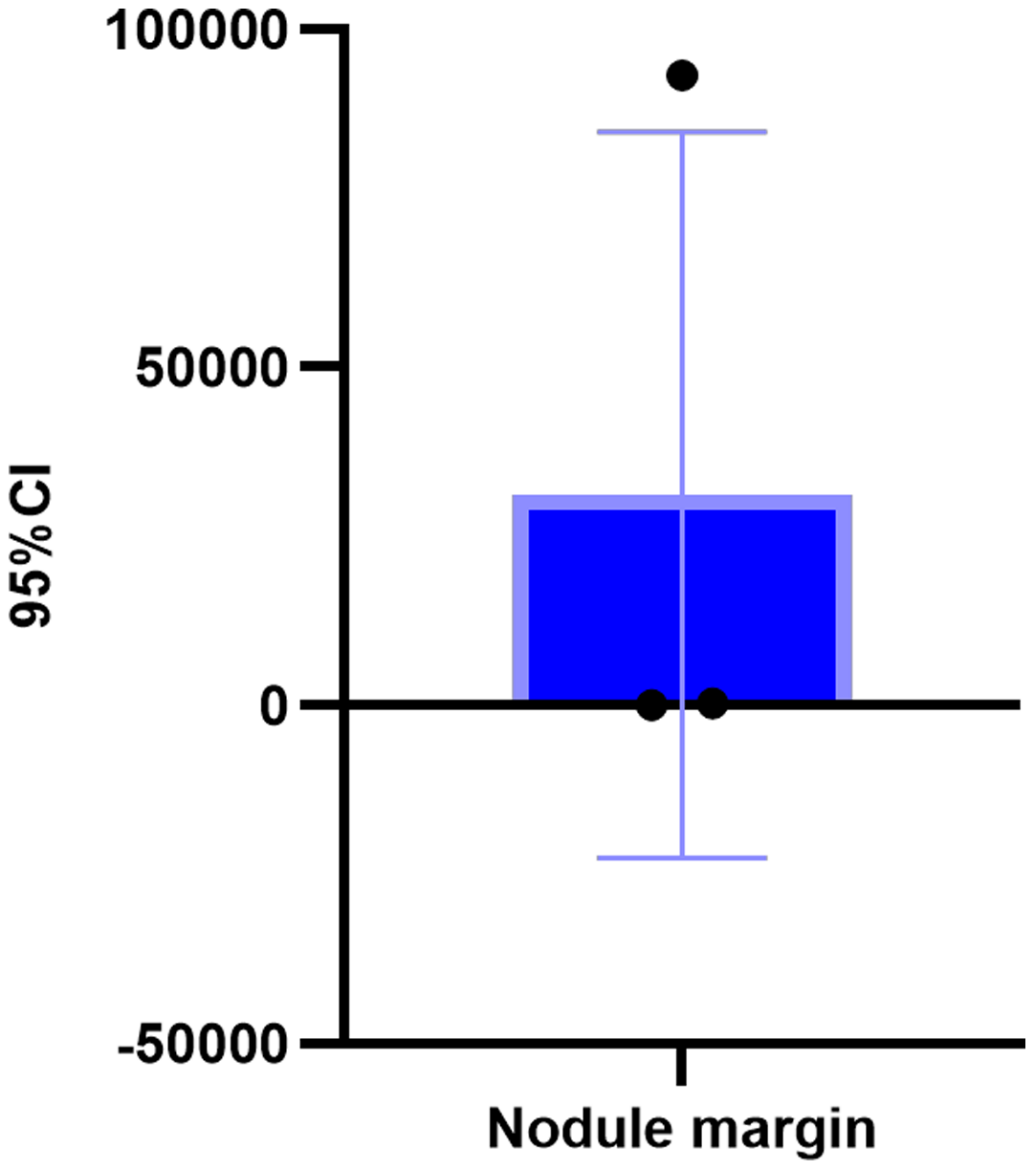
Binary logistic regression analysis of TN edge in C-TIRADS classification ultrasound features.

In a cohort of 303 patients, the HAMA results indicated that 126 patients exhibited normal anxiety levels, while 177 patients showed abnormal anxiety levels. Specifically, the HAMA results were categorized as follows: 126 patients without anxiety symptoms, 97 with possible anxiety, 47 with definite anxiety, 22 with definite and obvious anxiety, and 11 with possible severe anxiety. An ordinal logistic regression analysis was conducted to assess the impact of anxiety factors on c-tirads classification. These findings suggest that the presence of an unsmooth edge of TN is a risk factor for anxiety in patients, whereas protrusion outside the capsule is an influencing factor [*p* = 0.037, 0.028; *B* = 5.892, −5.723; OR, 95% CI: 362.080 (1.408–93142.282), 0.003 (0–0.054), Figures 4–7].

### Correlation analysis was conducted on the HAMA factors and CD-RISC resilience factors

**Figure 8:**
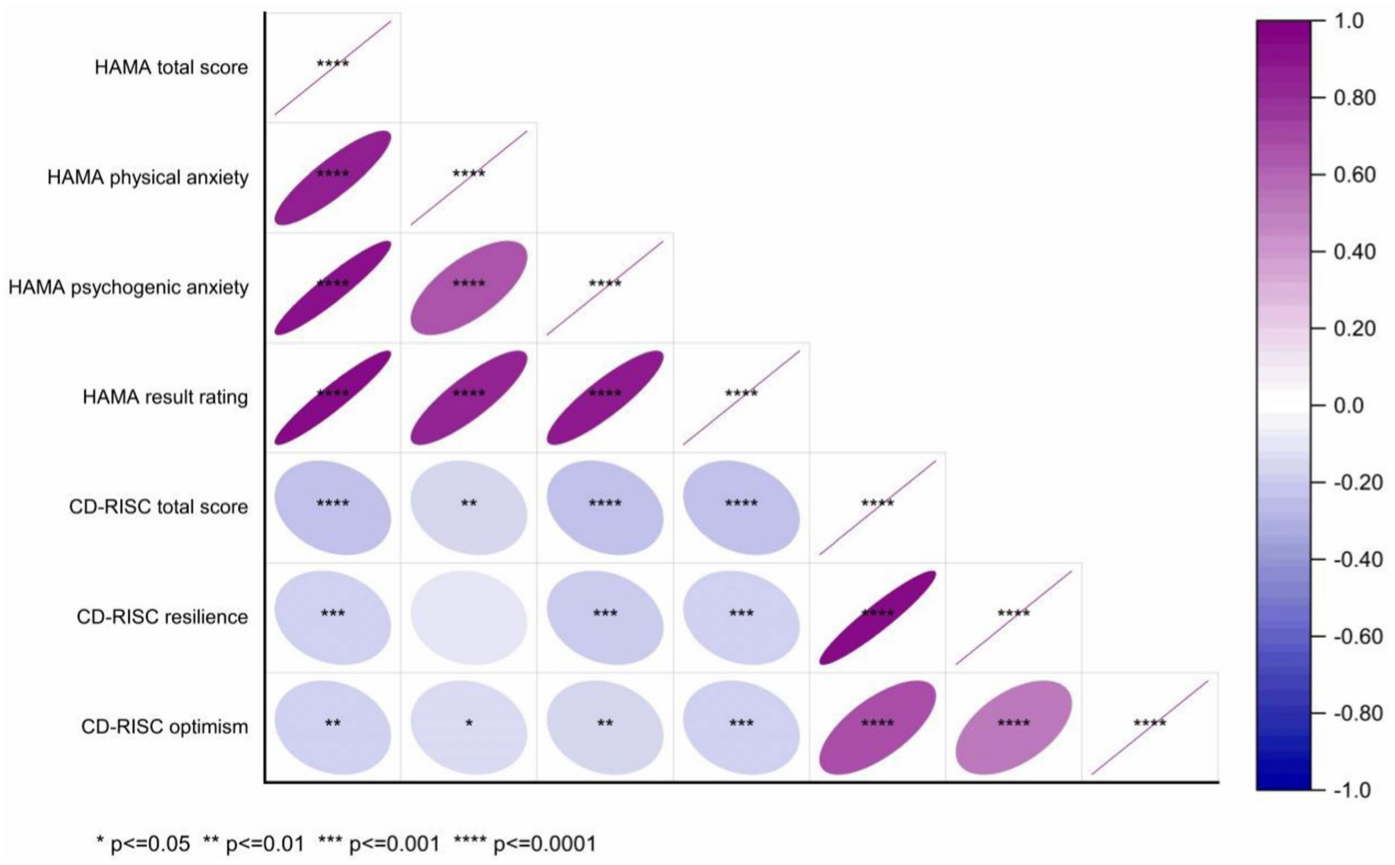
Spearman correlation analysis heatmap of HAMA factors and CD-RISC elasticity factors in TN (+) group.

These results indicate a Spearman correlation analysis was performed to examine the relationships between the factors of the Hamilton Anxiety Rating Scale (HAMA) and the resilience factors of the Connor-Davidson Resilience Scale (CD-RISC) (Figure 8). The correlation coefficients (*r*) between the HAMA total score and the CD-RISC total score, tenacity, and optimism were −0.241, −0.192, and −0.181, respectively, with significance levels of *p* < 0.001, *p* < 0.001, and *p* = 0.002. For the HAMA somatic anxiety factor, the correlation coefficients with the CD-RISC total score, tenacity, and optimism were −0.170, −0.109, and −0.146, respectively, with *p*-values of 0.003, 0.059, and 0.011. The correlations between the HAMA psychic anxiety factor and the CD-RISC total score, tenacity, and optimism were −0.248, −0.215, and −0.176, respectively, all significant at *p* < 0.001, *p* < 0.001, and *p* = 0.002. Finally, the correlation coefficients between the HAMA result rating and the CD-RISC total score, tenacity, and optimism were −0.259, −0.198, and −0.189, all significant at *p* < 0.001.

### Correlation analysis between HAMA and its 14 specific components

**Figure 9:**
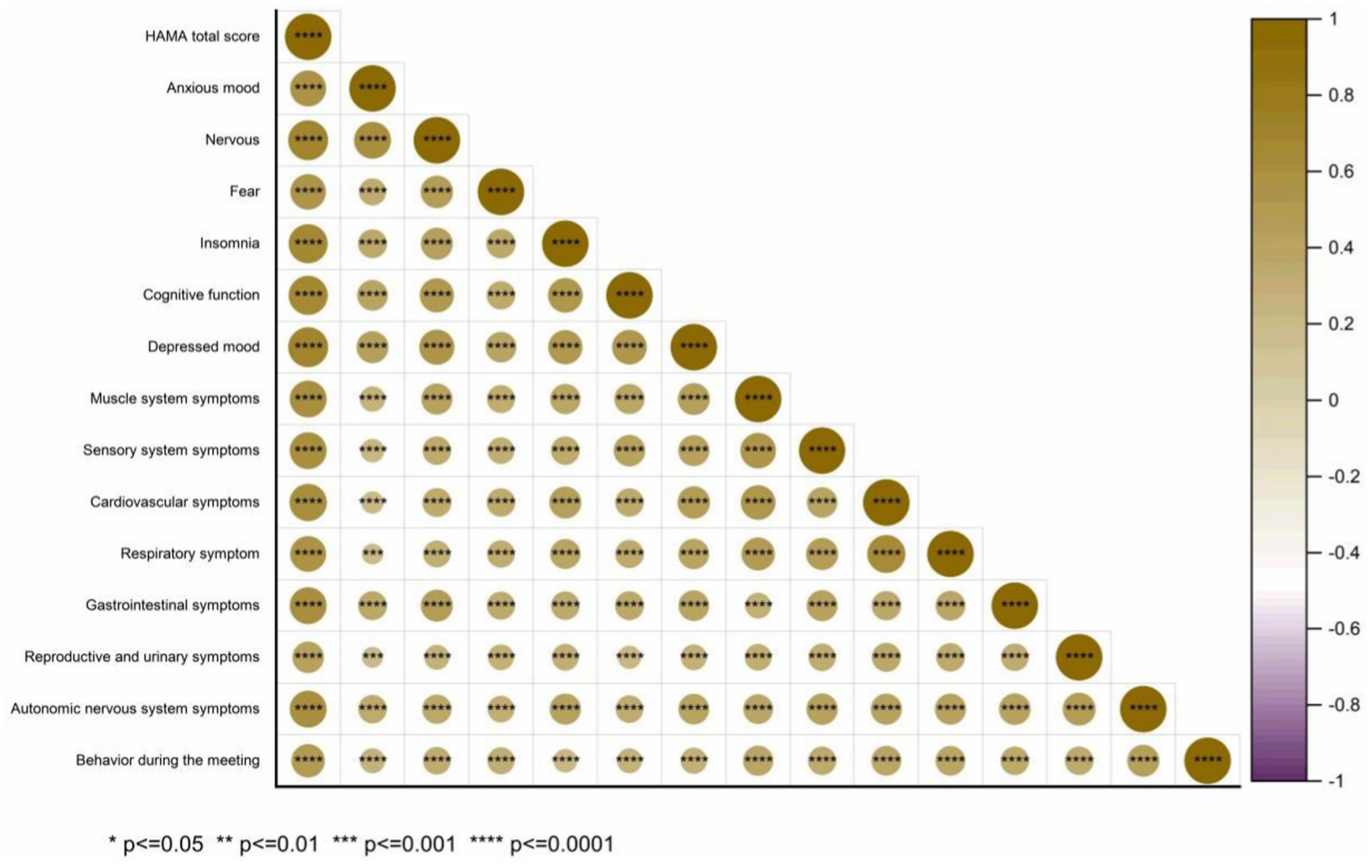
Hot map of correlation analysis between TN (+) group HAMA total score and HAMA 14 specific questions using Spearman.

**Figure 10:**
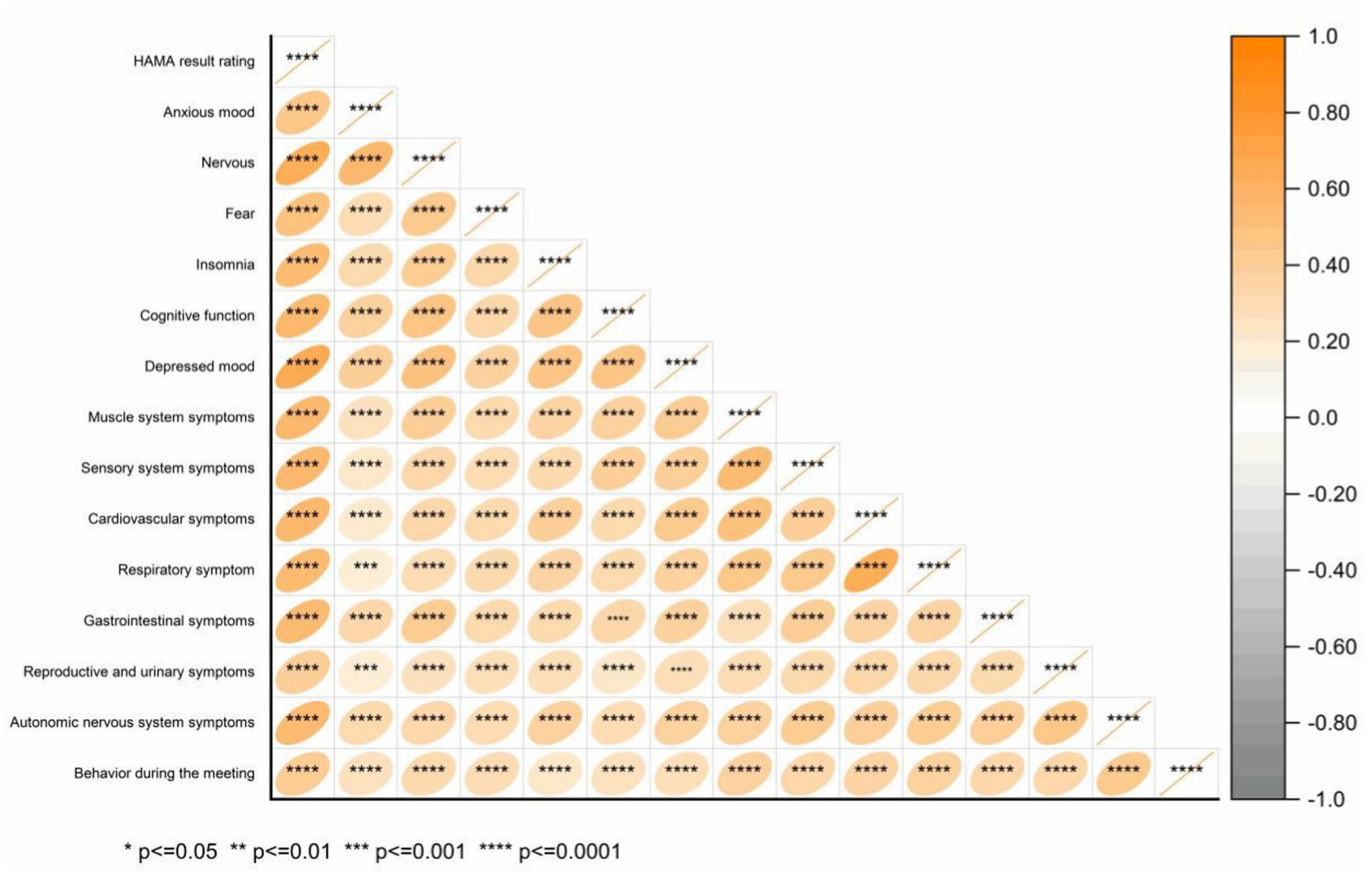
Hot map of correlation analysis between TN (+) group HAMA HAMA result rating and 14 specific questions using Kendal.

**Figure 11:**
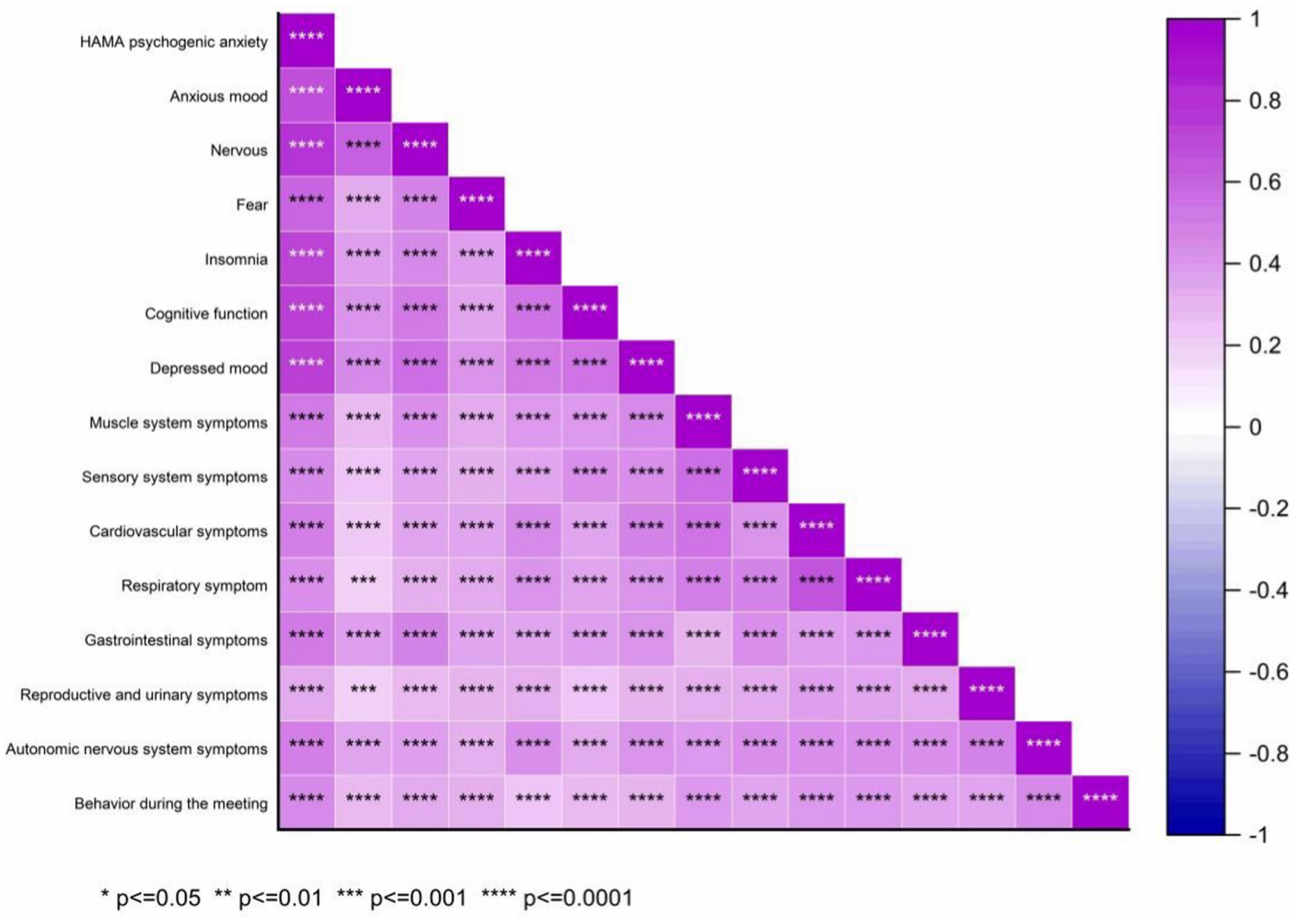
Hot map of correlation analysis between TN (+) group HAMA psychogenic anxiety and 14 specific questions using Spearman.

**Figure 12:**
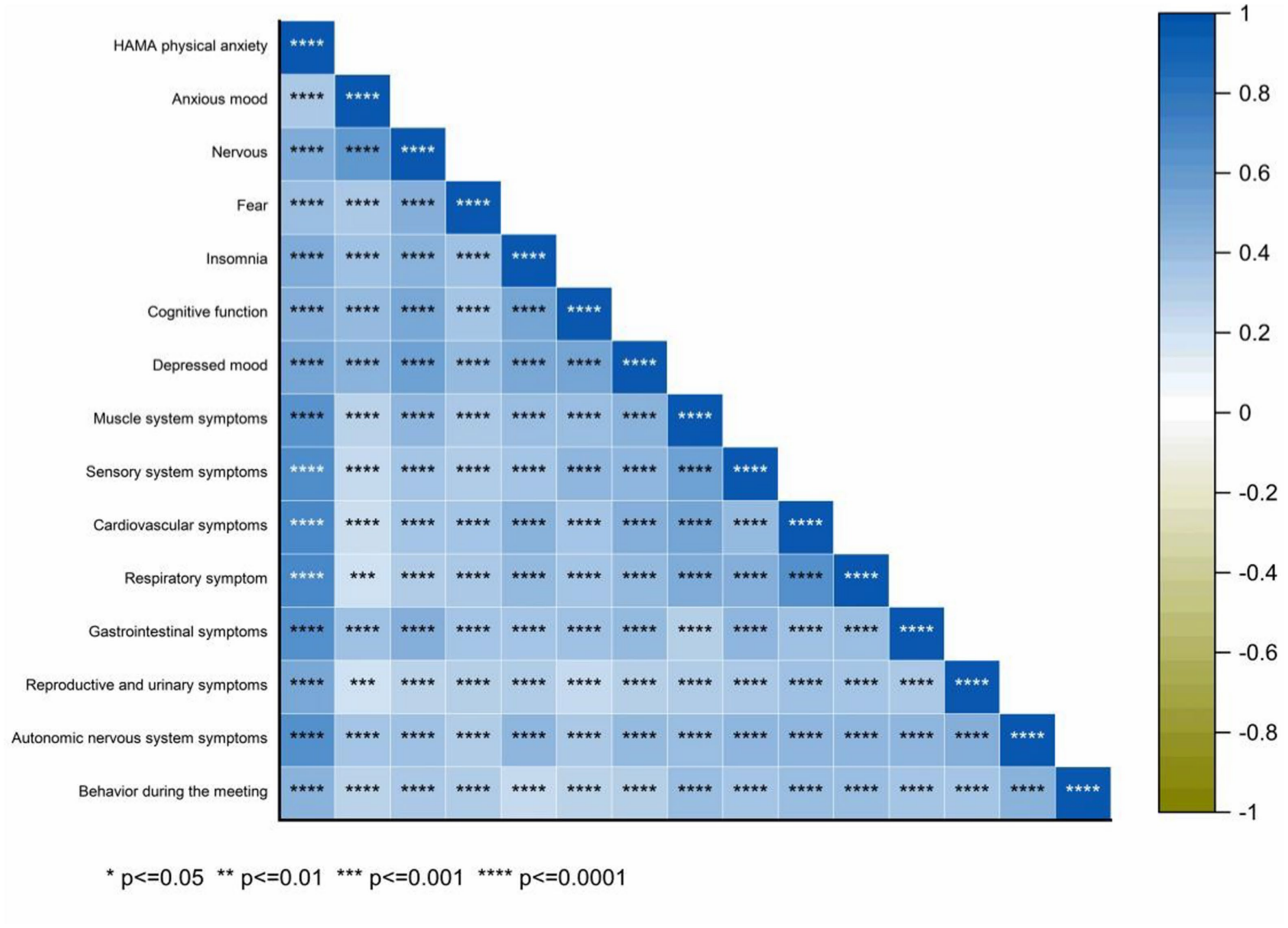
Hot map of correlation analysis between TN (+) group HAMA physical anxiety and 14 specific questions using Spearman.

Spearman correlation analysis was performed to explore the relationship between the HAMA total score and its 14 domains: anxiety, tension, fear, insomnia, cognitive function, depression, and symptoms of the muscular, sensory, cardiovascular, respiratory, gastrointestinal, reproductive, urinary, and autonomic nervous systems, as well as behavioral performance during meetings (Figure 9). The correlation coefficients (*r*) were: 0.598, 0.736, 0.574, 0.692, 0.698, 0.731, 0.624, 0.620, 0.637, 0.599, 0.625, 0.458, 0.629, and 0.507, all with *p* < 0.001. Kendall correlation analysis between the HAMA scores and 14 specific items (anxiety mood, tension, fear, insomnia, cognitive function, depressive mood, and symptoms of the muscular, sensory, cardiovascular, respiratory, gastrointestinal, reproductive, urinary, and autonomic nervous systems, as well as behavior during the interview) revealed *r* values of 0.480, 0.652, 0.512, 0.550, 0.568, 0.668, 0.576, 0.573, 0.576, 0.549, 0.542, 0.406, 0.554, and 0.438, respectively (all *p* < 0.001) (Figure 10). Spearman’s correlation analysis of HAMA somatic anxiety and 14 specific items (anxiety mood, tension, fear, insomnia, cognitive function, depressive mood, and symptoms of the muscular, sensory, cardiovascular, respiratory, gastrointestinal, reproductive, urinary, and autonomic nervous systems, as well as interview behavior) yielded *r* values of 0.355, 0.514, 0.412, 0.512, 0.497, 0.559, 0.652, 0.697, 0.703, 0.704, 0.664, 0.533, 0.680, and 0.476 (all *p* < 0.001) (Figure 11). Spearman’s correlation analysis of HAMA spiritual anxiety and 14 specific items (anxious mood, tension, fear, insomnia, cognitive function, depressive mood, and symptoms of the muscular, sensory, cardiovascular, respiratory, gastrointestinal, reproductive, urinary, and autonomic nervous systems, as well as interview behavior) yielded *r* values of 0.696, 0.795, 0.615, 0.712, 0.740, 0.757, 0.521, 0.480, 0.503, 0.454, 0.522, 0.356, 0.514, and 0.471 (all *p* < 0.001) (Figure 12).

## Discussion

Recent advancements in AI-assisted thyroid nodule diagnosis underscore the potential for integrating psychological metrics into multimodal risk assessment tools (Chen et al., 2023). Large-scale thyroid cancer studies emphasize the necessity of addressing both physical and mental health dimensions in patient-centered care (Yao et al., 2024). The ultrasound characteristics delineated by the C-TIRADS classification system can partially aid in predicting the benign or malignant nature of thyroid nodules (Street et al., 2024). Concurrently, psychological resilience is a pivotal factor in the management of thyroid nodules. Empirical evidence suggests that individuals with diminished psychological resilience exhibit heightened levels of anxiety, which may adversely influence the efficacy of disease treatment (Gao et al., 2022). The present study corroborates previous research, demonstrating that an increase in the C-TIRADS classification correlates with a decrease in psychological resilience among patients with thyroid nodules. Furthermore, reduced psychological resilience is linked to a higher probability of presenting with a solitary thyroid nodule. This association implies that individuals with lower psychological resilience are more susceptible to physiological responses in the endocrine system, particularly the thyroid, when confronted with life stressors and emotional challenges (Pitt et al., 2021; Abualruz et al., 2024). The thyroid gland, a critical component of the endocrine system, is notably influenced by psychological conditions (Phillips et al., 2013). When an individual’s psychological resilience is diminished, prolonged mental tension and emotional fluctuations can lead to elevated levels of stress hormones in the body. This hormonal imbalance may disrupt the normal functioning of the thyroid gland via the hypothalamus-pituitary-thyroid (HPT) axis (Klein, 2021). Chronic stress can result in abnormal proliferation and differentiation of thyroid cells, potentially leading to the formation of nodules.

Furthermore, the detection of TN often induces anxiety and concern among patients. Nodules with irregular edges are frequently perceived as having invasive or malignant potential. Upon learning that their nodules possess irregular edges, patients may associate this with the likelihood of malignant lesions, thereby experiencing heightened psychological stress and anxiety (Lei et al., 2023). In this study, the protrusion of the nodule beyond the thyroid capsule was considered a protective factor. This may be attributed to the perception that such nodules are generally easier to monitor and treat, which can reassure patients that the condition is manageable, thereby alleviating fears related to the progression of unknown diseases.

The study identified a negative correlation between the anxiety levels of patients with thyroid nodules and their resilience. Specifically, the findings revealed a low to very low degree of negative correlation between the HAMA total score and the CD-RISC total score, as well as between resilience and optimism. This suggests that as anxiety levels increase, the resilience of patients, along with its various dimensions, tends to decrease. These results underscore the importance of addressing not only the physiological symptoms but also the mental health of patients with thyroid nodules (Figure 8). The observed low negative correlation between the HAMA total score and the CD-RISC total score indicates that patients exhibiting severe anxiety symptoms generally possess lower overall resilience. This phenomenon may be attributed to the fact that anxiety symptoms heighten patients’ concerns about their health status, thereby diminishing their confidence in managing their condition and consequently reducing their psychological resilience. The findings indicate an extremely low correlation between the Hamilton Anxiety Rating Scale (HAMA) somatic anxiety factor and the Connor-Davidson Resilience Scale (CD-RISC) total score, resilience, and optimism, suggesting that somatic anxiety symptoms exert minimal influence on resilience dimensions. This may be attributed to the nature of somatic anxiety symptoms, which typically manifest as physical discomforts such as palpitations and sweating, leading to unease and discomfort (Singh et al., 2024), yet exerting negligible impact on the core components of psychological resilience. Conversely, the low negative correlation between the HAMA psychogenic anxiety factor and the CD-RISC total score, resilience, and optimism indicates that psychogenic anxiety symptoms have a more pronounced effect on patients’ resilience. Psychogenic anxiety symptoms, characterized by excessive worry, tension, and fear, not only disrupt daily life but may also undermine psychological resilience (Evans et al., 2024). Furthermore, the low negative correlation between the overall HAMA score and the CD-RISC total score, resilience, and optimism underscores the differential impact of anxiety levels on resilience dimensions. Generally, an inverse relationship exists between anxiety levels and resilience, particularly concerning the dimensions of resilience and optimism. This observation indicates that enhancing patients’ resilience and optimism could be crucial for improving their overall resilience. Tenacity represents a patient’s perseverance and persistence when confronted with pressure and challenges, whereas optimism denotes a patient’s positive expectations and confidence regarding the future (Long et al., 2024; Yang et al., 2023). Consequently, clinical interventions should not only aim to alleviate patients’ anxiety but also focus on bolstering their resilience and optimism through positive psychological strategies, thereby strengthening their overall psychological resilience.

The anxiety experienced by patients with TN exhibits varying correlations across multiple dimensions. Notably, the HAMA total score demonstrated a significant positive correlation with tension and depression, indicating a strong association between anxiety symptoms and both tension and depression in TN patients. This finding underscores the importance for clinicians to carefully consider the emotional states of tension and depression when assessing and managing patients with thyroid nodules. Regarding somatic anxiety, the HAMA somatic anxiety score showed a strong correlation with cardiovascular and respiratory symptoms, suggesting that in TN patients, somatic anxiety primarily manifests through these symptoms. Consequently, healthcare professionals should closely monitor the cardiovascular and respiratory health of TN patients experiencing anxiety and may need to implement targeted physical examinations and interventions.

In the context of mental anxiety, the Hamilton Anxiety Rating Scale (HAMA) psychic anxiety score demonstrates a robust correlation with tension, insomnia, cognitive function, and depressive states. This suggests that psychic anxiety in patients with TN manifests as a complex interplay of various psychological symptoms. It is important for healthcare providers to consider these factors when developing treatment plans for TN patients with anxiety. These findings underscore the importance of adopting a comprehensive and multidimensional approach to understanding the psychological state of patients during mental health assessments, rather than concentrating solely on isolated symptoms. Additionally, the correlation analyses elucidate the characteristics and interrelationships among different types of anxiety in TN patients. Specifically, the total HAMA score exhibits a moderate correlation with anxiety-related emotions, fear, and insomnia—factors that contribute to the overall presentation of anxiety but are not predominant. Conversely, the HAMA psychic anxiety score shows a stronger correlation with insomnia and cognitive function, indicating that these symptoms may be more pronounced in the context of psychic anxiety. From a clinical intervention perspective, these findings provide a foundation for the development of personalized treatment plans tailored to the unique psychological profiles of TN patients. For patients with TN who exhibit pronounced tension and depression, psychotherapy should be prioritized to address these emotional symptoms (Zhang et al., 2023). Conversely, for patients experiencing somatic anxiety characterized by cardiovascular and respiratory manifestations, a comprehensive approach that includes pharmacological treatment and physical health management may be required to mitigate these physical symptoms (Romanazzo et al., 2022).

### Limitations

The Hamilton Anxiety Rating Scale (HAMA) can partially reflect an individual’s anxiety level; however, its sensitivity and accuracy vary across different specific symptoms. The individual items within the HAMA scale address diverse aspects of anxiety symptoms, which can significantly differ among individuals. For instance, some patients may predominantly exhibit physical symptoms such as muscle tension and gastrointestinal discomfort, whereas others may present with more psychological symptoms, including anxiety and cognitive impairment. Therefore, when employing the HAMA scale for evaluation, it is imperative to conduct a comprehensive analysis in conjunction with specific symptoms. Furthermore, the moderate correlation observed suggests that the HAMA scale may not fully capture subtle or specific changes in anxiety symptoms. Although there is some association between the autonomic nervous system symptoms and behavioral performance of patients with anxiety disorders and their HAMA scores, this correlation is not sufficiently robust. This may indicate that the weighting of these symptoms within the scale is inadequate or that the scoring criteria lack precision. Moreover, this study also has limitations: (1) cross-sectional design precludes causal inference; (2) gender imbalance (87.5% female) limits generalizability; (3) not account for socioeconomic status or general health anxiety, which may confound psychological outcomes; (4) pre-examination baseline assessments would better distinguish pre-existing anxiety from diagnostic reactions. Future studies should adopt this approach.

## Conclusion

The study found ultrasonic characteristics of C-TIRADS revealed significant variations in TN across different malignant risk stratifications. Higher malignant risk in TN was associated with reduced psychological resilience in patients, and such nodules were more likely to present as solitary. An irregular nodule edge was identified as a risk factor for anxiety. Resilience was found to be negatively correlated with anxiety levels, with patients exhibiting severe anxiety symptoms demonstrating lower resilience, particularly in terms of toughness and optimism. Mental anxiety profoundly impacts the psychological state of patients, underscoring the importance of addressing mental health in the management of TN patients. Targeted psychological interventions, combined with C-TIRADS-based risk stratification, may improve resilience and reduce anxiety in thyroid nodule patients, ultimately enhancing treatment adherence and quality of life. This study contributes to refining the diagnostic and therapeutic strategies for thyroid nodules, enabling clinicians to develop more personalized treatment plans and ultimately enhancing patients’ quality of life and treatment outcomes.

## Data Availability

The original contributions presented in the study are included in the article/supplementary material. Further inquiries can be directed to the corresponding authors.
